# Delineating the molecular responses of a halotolerant microalga using integrated omics approach to identify genetic engineering targets for enhanced TAG production

**DOI:** 10.1186/s13068-018-1343-1

**Published:** 2019-01-04

**Authors:** Neha Arora, Poonam Kumari, Amit Kumar, Rashmi Gangwar, Khushboo Gulati, Parul A. Pruthi, Ramasare Prasad, Dinesh Kumar, Vikas Pruthi, Krishna Mohan Poluri

**Affiliations:** 1 Department of Biotechnology, Indian Institute of Technology Roorkee, Roorkee, Uttarakhand 247667 India; 2 Centre for Transportation Systems, Indian Institute of Technology Roorkee, Roorkee, Uttarakhand 247667 India; 3 Centre of Biomedical Research, SGPGIMS, Lucknow, Uttar Pradesh 226014 India

**Keywords:** Microalga, TAG production, *Scenedesmus* sp. IITRIND2, Halotolerant, Algal-omics, Genetic engineering, Sea water

## Abstract

**Background:**

Harnessing the halotolerant characteristics of microalgae provides a viable alternative for sustainable biomass and triacylglyceride (TAG) production. *Scenedesmus* sp. IITRIND2 is a fast growing fresh water microalga that has the capability to thrive in high saline environments. To understand the microalga’s adaptability, we studied its physiological and metabolic flexibility by studying differential protein, metabolite and lipid expression profiles using metabolomics, proteomics, real-time polymerase chain reaction, and lipidomics under high salinity conditions.

**Results:**

On exposure to salinity, the microalga rewired its cellular reserves and ultrastructure, restricted the ions channels, and modulated its surface potential along with secretion of extrapolysaccharide to maintain homeostasis and resolve the cellular damage. The algal-omics studies suggested a well-organized salinity-driven metabolic adjustment by the microalga starting from increasing the negatively charged lipids, up regulation of proline and sugars accumulation, followed by direction of carbon and energy flux towards TAG synthesis. Furthermore, the omics studies indicated both de-novo and lipid cycling pathways at work for increasing the overall TAG accumulation inside the microalgal cells.

**Conclusion:**

The salt response observed here is unique and is different from the well-known halotolerant microalga; *Dunaliella salina*, implying diversity in algal response with species. Based on the integrated algal-omics studies, four potential genetic targets belonging to two different metabolic pathways (salt tolerance and lipid production) were identified, which can be further tested in non-halotolerant algal strains.

**Electronic supplementary material:**

The online version of this article (10.1186/s13068-018-1343-1) contains supplementary material, which is available to authorized users.

## Introduction

Generation of sustainable clean alternatives to fossil fuels is one of the most daunting challenges confronting the global energy fulfillment and in turn the economic prosperity. Lipid-rich microalgae can play a central role in reducing the burden on fossil-based fuels because of their high areal yields and possibility of cultivation on non-arable lands [1]. However, despite the consistent efforts, high costs of production have derailed the commercialization of microalgal-based biodiesel. The three key factors that could expedite the economical production of biodiesel production include the following: (a) increasing the algal biomass with low-cost feedstocks, (b) efficient extraction and conversion of lipids to biodiesel, and (c) decreasing the usage of fresh water [2–4]. Utilization of saline water (sea water or saline groundwater) can be a sustainable feedstock for large-scale cultivation of microalgae (either in open ponds or closed photobioreactors) due to its high abundance, containment of essential nutrients, and less chances of contamination, along with reduction of load on fresh water reserves [5, 6]. Sea water can also be utilized for process cooling which can further reduce the fresh water consumption [4]. Apart from the above-listed advantages, cultivation of microalgae in sea water also results in a high lipid content, easier extraction of lipids, resistance to contamination, and increase in the settling efficiency (attributed to floc formation and large cell size) making the downstream processing economically feasible [7].

However, to exploit sea water as cultivation medium, the bioprospecting or generation of halotolerant species with high biomass and lipid productivity is imperative [7, 8]. Halotolerant microalgae have the inherent ability to adapt and thrive in high saline conditions [9]. Salt tolerance by microalgae is a complex phenomenon involving a plethora of morphological, biochemical, and molecular changes that aid its survival. These microalgae can regulate their ion transport, modulate membrane permeability, synthesize osmolytes and other stress-related molecules that restore the turgor pressure, and protect the cell from salinity-generated reactive oxygen species (ROS), thereby adjusting to the new environment [8, 10, 11].

Salt tolerance by microalgae can be divided into three stages: alarm response, regulation, and acclimatization [12]. Alarm response involves the influx of water into the microalga cells, resulting in increase in cell size and volume [12, 13]. The resultant algal cells balance the internal osmotic pressure by accumulation of osmolytes and active efflux of Na^+^ ions. The regulation phase involves post-translational modifications, activation/deactivation of light harvesting complexes (LHC), enzymes, and transporters. Recently, downregulation of photosystem II (PSII), antenna proteins, and chlorophyll content was reported in *Scenedesmus obliquus* XJ002 and *Chlorella* sp. S30 under high saline conditions [14, 15]. The acclimatization stage includes changes in the translational control of genes and proteins, and activation of Ca^2+^ signaling [12, 16]. This process ensures protection of microalga cells under saline stress by renaturation of damaged cellular components and initiation of antioxidant mechanism [16]. Accumulation of antioxidant enzymes, osmolytes, and osmoprotectant molecules has been reported in *Neochloris oleoabundans* and *Chlorella* sp. S30, when cultivated under NaCl stress [15, 17].

Technological advances in “algal-omics” approaches such as transcriptomics, proteomics, metabolomics, and lipidomics on various microalgal strains under different conditions have strengthened our understanding of microalgal’s response and adaptation towards both biotic and abiotic stress [18]. These integrated omics approaches not only depict the underlying molecular mechanisms responsible for microalgal strain adaptability under a given stress condition, but also identifies new pathways, and genetic targets to engineer novel strains with desired characteristics. Transcriptomics studies on *N. oleoabundans* and *Picochlorum* sp. evidenced for an upregulation of osmolytes, antioxidant, and carbon concentration mechanism (CCM) genes for their survival under saline environments [11, 14]. Several of the previous studies mainly concentrated on the results of lipid accumulation of microalgae cultivated in seawater/saline water, while little information is available concerning the metabolic mechanism behind the improvements in lipid accumulation through using integrated omics approach.

Recently, we reported the halotolerance characteristics, and high lipid accumulation capability of a novel fresh water microalga *Scenedesmus* sp. IITRIND2 when cultivated in artificial sea water (ASW) [9]. The microalga adapted to saline conditions by altering its biochemical composition (increase in lipids), accumulation of osmolytes, and bolstering its antioxidant activity to quench the ROS. Understanding the halotolerance mechanism of such fresh water microalgal strain(s) could be helpful in gaining insights into the key regulatory pathways that could facilitate future metabolic engineering strategies to leverage successful manipulation of non-halotolerance oleaginous microalgae into high lipid accumulating halotolerant strains. Hence, to gain mechanistic insights into the halotolerant nature of this unique microalga, integrated omics studies are imperative.

The present work aims to delineate the metabolic pathway interactions and regulatory genes involved in adaptation of a microalga (*Scenedesmus* sp. IITRIND2) in response to full-strength sea water as compared to fresh water. The physiological changes in the microalga in response to salinity have been studied using electron microscopy, zeta potential, and extracellular polysaccharide (EPS) formation. The study also sheds light into the changes in the membrane permeability by analyzing key intracellular ions (Na^+^, Mg^2+^, Ca^2+^, and K^+^). Furthermore, differential protein, metabolite, and lipid expression profiles were obtained using metabolomics, proteomics, and lipidomics. The study provides baseline for applying metabolic engineering approaches to generate wide variety of halotolerant microalgal species.

## Materials and methods

### Microalgae cultivation

The microalga *Scenedesmus* sp. IITRIND2 (Genebank Accession no. KT932960) was previously isolated from a fresh water lake as reported elsewhere and maintained in Bold’s Basal media [20]. The microalga was adapted to artificial sea water (ASW) as described elsewhere [9]. These adapted cultures were then used for further studies by cultivating in 250 mL Erlenmeyer flasks for a period of 7 days.

### Analysis of intracellular metal ions and zeta potential of microalga

The intracellular ions (Na^+^, Mg^2+^, Ca^2+^, and K^+^) of the cultivated cells in ASW and control medium were extracted using the method described by Wiley et al., and estimated using inductive coupled plasma mass spectroscopy (ICP-MS; Perkin-Elmer, ELAN DRC-e) [21]. The zeta potential (ZP) of the microalgal cells was obtained at 25 °C and in suspension under an applied electric field of 80 mV using a zeta sizer (Nano-Z590, Malvern). The zeta potential of microalgal cells was measured using the Malvern software (v 7.03).

### Electron microscopy of microalgal cells

Fourier electron scanning electron microscopy (FE-SEM) using our earlier optimized protocol was performed to analyze the size and surface of microalgal cells [20]. Transmission electron microscopy (TEM) was performed to visualize the ultrastructure of cells. Briefly, microalgal cells grown in control and ASW were harvested on the 7th day, and then fixed overnight with 2.5% glutaraldehyde and 2% paraformaldehyde in 0.1 M phosphate buffer (pH 7.4) at 4 °C. The cell pellets were then washed thrice with 0.1 M phosphate buffer and post-fixed with osmium tetraoxide (OsO_4_) in Soresen phosphate buffer (0.05 M buffer, 1% OsO_4_, and 0.25 M glucose) for 1 h and again washed twice with distilled water. The cells were double stained with uranyl acetate and lead citrate solution for 12 h, and then visualized under TECNAI 200 kV TEM (Fei, Electron Optics).

### Proton NMR-based metabolomics and lipidomic analysis

The metabolites from the microalgal cells cultivated in ASW and control were extracted using our earlier established protocol from the cultures harvested on the 7th day [22]. To these samples, 550 μL of D_2_O containing a chemical shift indicator (4,4-dimethyl-4-silapentane-1-sulfonic acid (DSS), 0.5 mM) was added and the proton (^1^H) CPMG (Carr–Purcell–Meiboom–Gill) NMR spectra were then acquired on a 800 MHz NMR spectrometer (Bruker Avance III) equipped with Cryoprobe. The raw NMR spectral data were processed using standard Fourier transformation (FT) procedure in the Bruker software Topspin-v2.1. Prior to FT, each free induction decay (FID) was zero-filled to 64 k data points, multiplied by an exponential window function, and a line-broadening function of 0.3 Hz was applied. Chemical shifts in the spectra were identified and assigned for various metabolites using commercial software Chenomx NMR Suite (form Chenomx Inc., Edmonton, AB, Canada containing 800 MHz chemical shift database).

The multivariate data analysis was performed by integrating the NMR spectra of six biological replicates of each culture and normalized against the internal standard (DSS) at 0 ppm to obtain NMR-based metabolite profiling. Prior to multivariate data analysis, all the 1D ^1^H CPMG NMR spectra were manually phased, baseline corrected, and referenced internally to the methyl resonance of lactate at *δ* 1.3102. The data were then reduced into spectral bins (0.03 ppm width) using Pathomx [23]. The spectral bins were then imported into Metaboanalyst (v3.0) software and scaled to Pareto variance for multivariate analysis [24, 25]. Principal component analysis (PCA) was performed and metabolites of discriminatory significance were identified in the 2D loading plot based on their coefficient score > ± 0.1. The discriminatory metabolites were further tested for statistical significance using student *T* test in Metaboanalyst. The relative metabolic changes were assessed using unsupervised hierarchical clustering using Ward linkage which was further employed to create the heat map consisting of 25 metabolite entities (with *p* < 0.001) that had the highest impact on separation of the different treatment groups.

For lipidomic analysis, the total lipid was extracted using the modified Bligh and Dyer method [20]. The total extracted lipids (10 mg) were mixed with 550 μL deuterated chloroform (CDCl_3_) and ^1^H NMR spectra were recorded using a 500 MHz NMR spectrometer. The chemical shifts in the spectra were identified and assigned using earlier published studies [26]. The intensity/fold change in their respective peaks was obtained using Burked Topspin 3.5.

### Proteomic analysis using mass spectrometry

For protein extraction, ASW and control grown cultures were harvested on the 7th day by centrifuging at 4000×*g* for 10 min at 4 °C. The cell pellets (1 g fresh DCW) were washed thrice with chilled double distilled water and then frozen at − 80 °C. The total protein was extracted using the protocol of Guarnieri et al. [27] with minor modifications. In detail, the cell pellets were grounded in liquid nitrogen and then solubilized on ice in 1 mL lysis buffer [50 mM Tris; pH 8.0; 150 mM NaCl, 1 mM DTT, and 10% glycerol supplemented with 1× complete protease inhibitor cocktail (Roche Diagnostics Corporation, Indianapolis, IN)]. The process was repeated twice, and then, the supernatant was collected for proteomic analysis. Total soluble protein content was quantified using Bradford assay, and 20 μg of soluble protein was resolved using 12% SDS-PAGE. Three biological replicates were used for protein isolation and all subsequent analysis. The selected protein bands were then manually excised and washed with 25 mM ammonium bicarbonate in 40% acetonitrile, followed by reduction with 10 mM DTT in 20% acetonitrile for 20 min at room temperature. The gel bands were then alkylated with 40 mM iodoacetamide in 20% acetonitrile for 15 min at room temperature. Gel bands were further washed as described above followed by incubation in 100% acetonitrile on ice for 10 min. The proteins were then digested overnight at 37 °C by adding 25 μg/mL trypsin (Gold trypsin, Promega, Madison, WI) in 30 mM acetonitrile. The resulting peptide mixture was acidified by addition of 0.1% formic acid for 20 min and then dried in vacuum concentrator and stored at − 20 °C till further processing.

Protein identification was performed on MALDI-TOF–MS/MS (Autoflex, speedTM, Bruker Dalton, Bremen, Germany). Equal volumes of trypsinized protein fragments and matrix solution containing 5 mg/mL of α-cyano-4 hydroxycinnamic acid (Sigma Aldrich Fluka, St. Louis, MO) were prepared in trifluoroacetic acid 40 (600 μL MS grade water, 400 μL acetonitrile, and 0.6 μL trifluoroacetic acid). The mixture was spotted on a 96-well MALDI-TOF–MS target plate with peptide mix (Brukers Dalton, Bremen, Germany) as calibrant. The spectra were collected from 300 shots per spectrum over *m*/*z* range of 700–3000 and the peak list was generated using Flex analysis 3.0. All the MS/MS spectra were searched against Chlorophyta protein database from the Uniprot website (http://www.uniprot.org/taxonomy/3041) using the MASCOT search engine version 2.2 (Matrix Science) with MW, pI, modifications: carboamidomethyl (C), variable modifications; acetyl (N term), oxidation (Met), and a mass tolerance of ± 0.1 Da. Mascot score greater than 55 was considered significant. The up/down regulation of proteins was estimated using the Image J 1.4a software.

### RNA extraction and quantitative qRT-PCR analysis

Total RNA was extracted from the harvested microalgal biomass using RNeasy plant mini kit (Qiagen) according to the manufacturer’s instructions. The yield of the total RNA extraction was measured by NanoDrop spectrophotometer (Biorad). For real-time RT-PCR analysis, the first-strand cDNA synthesis was carried out from 1 μg of total RNA using Verso cDNA synthesis kit (Thermo Scientific) according to the manufacturer’s instructions. Gene-specific primers were designed to amplify fragments of 100–150 bp in length (Additional file 1: Table S1). *Scenedesmus* sp. β-actin primers were used to demonstrate the equal amounts of templates and loading. RT-PCR was carried out on Eppendorf RT-PCR. Gene expression was calculated using 2^−ΔΔ*Ct*^ method [Δ*Ct* = *Ct* (target gene) − *Ct* (house-keeping gene)] where *Ct* is the cycle number at which the fluorescent signal rises statistically above the background [28].

### Compositional analysis of extracellular polysaccharide (EPS)

The EPS was extracted by harvesting the ASW and control cultures at 15,000*g* for 20 min; the supernatant was filtered (Whatmann, UK) twice and then concentrated to one-fourth volume on magnetic stirrer at 60 °C for 12 h [29]. The EPS was precipitated by gradually mixing equal volume of cold methanol to the supernatant and then kept at 4 °C overnight. The supernatant was then centrifuged at 15,000*g* for 30 min at 4 °C to remove methanol, washed with absolute ethanol, and then dissolved in Milli-Q water (Millipore, USA). The EPS was then dialysed against distilled water for 24 h using 1 kD membrane and lyophilized.

Total carbohydrates and proteins in the lyophilized EPS were estimated using phenol–sulphuric method and Bradford assay, respectively [30]. Fourier transform infrared spectroscopy (FT-IR) spectra of EPS were recorded in the region of 4000–400 cm^−1^ on a GX FT-IR spectrometer (Perkin-Elmer, USA).

### Statistical analysis

All the experiments were performed in triplicates (except for metabolomics which had six replicates) and the results have been presented as mean ± S.D. One-way ANOVA followed by *T* test was done for statistically significant results with *p* < 0.05.

## Results

In the face of high salinity, halotolerant microalgae undergo a series of adaptive changes both at physiological and molecular levels. Deciphering the salinity response at both levels is crucial not only to understand the algal physiology but also to unveil potential genome editing targets for tailor-made high lipid accumulating halotolerant microalgal strains. In this investigation, we evaluated various physiological (ion transport, membrane potential, ultrastructure, and EPS) and molecular (metabolomics, proteomics, and lipid composition) adaptation aspects of *Scenedesmus* sp. IITRIND2, a prospective high lipid and halotolerant strain for biodiesel production.

### Salinity-induced changes in intracellular ion composition and membrane potential of *Scenedesmus* sp. IITRIND2

**Fig. 1:**
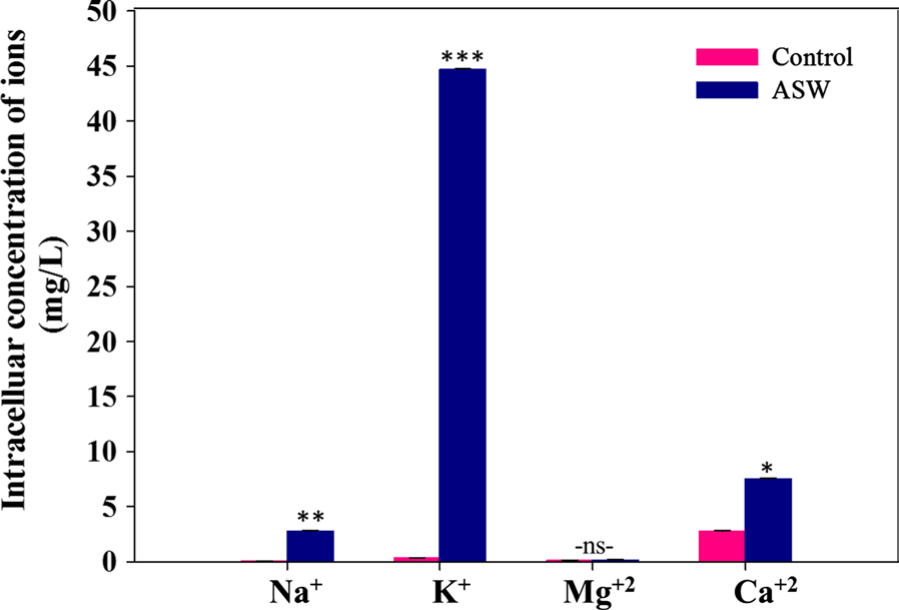
Intracellular concentration of ions in control and ASW cultures of *Scenedesmus* sp. IITRIND2 analyzed on the 7th day

The first line of defense deployed by the microalga to adapt and thrive in high saline environments is selective retention or exclusion of ions. Monovalent ions such as Na^+^, K^+^, and divalent ions including Mg^2+^ and Ca^2+^ are crucial for maintaining the turgor pressure and osmotic balance inside the cells [31]. Analysis of the intracellular ions of *Scenedesmus* sp. IITRIND2 cultivated in ASW showed maximum concentration (44.7 ± 0.6 mg/L) of K^+^ which was ~ 100-fold higher as compared to control (Fig. 1). Interestingly, despite high Na^+^ concentration in the ASW (23 g/L), its intracellular concentration was 2.8 ± 0.1 mg/L, being ~ 40-fold higher than control which suggests active extrusion of Na^+^ from the cells against the concentration gradient (Fig. 1). Earlier, salinity tolerance in *Dunaliella salina* is reported to be mediated by up regulation of two distinct Na^+^ extrusion systems in the plasma membrane including a Na^+^ ATPase and NADH-driven electron transport Na^+^ pump (H^+^ ATPase pump) [32]. On the other hand, intracellular concentration of Ca^2+^ in ASW-grown cells was only ~ 2.6-fold higher than control cultures, while no statistical difference was recorded in Mg^2+^ concentration (Fig. 1). The data suggested that K^+^ is the major osmolyte that competed and regulated the accumulation of toxic Na^+^ inside the microalgal cells. Similar results have been also reported in *Chlorella pyrenoidosa* and *Prasiola crispa* when cultivated in high saline medium [33, 34]. Furthermore, a high K^+^/Na^+^ ratio inside the cells has been reported to increase the salinity tolerance in plants and could be the same for salt-tolerant microalgae [35].

Selective K^+^ retention in the ASW-cultivated cells can alter the membrane potential for maintaining the turgor pressure. Hence, we measured the surface zeta potential of the cells under halotolerant conditions. The observed zeta potential of *Scenedesmus* sp. IITRIND2 grown in control medium was − 6 mV which decreased to − 12 mV in ASW-cultivated cells. A similar decrease in zeta potential has also been observed for *Chlorella pyrenoidosa* when grown in NaCl medium [31].

### Morphology and ultrastructure changes in *Scenedesmus* sp. IITRIND2 under halotolerant conditions

**Fig. 2:**
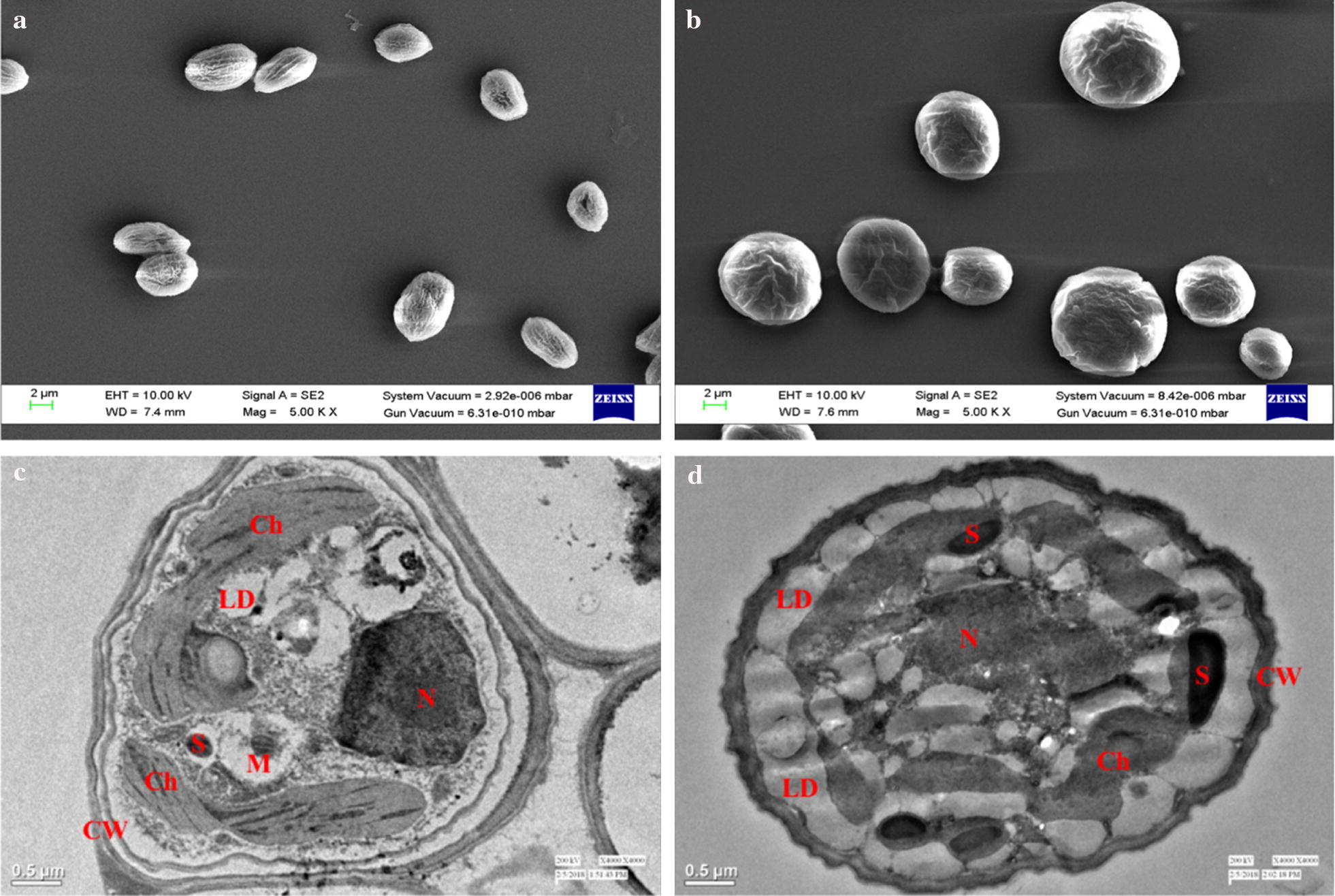
Electron micrographs of *Scenedesmus* sp. IITRIND2: **a** FE-SEM of control, **b** FE-SEM of ASW, **c** TEM of control, and **d** TEM of ASW cells on the 7th day. Chloroplast (Ch), nucleus (N), mitochondria (M), starch granules (S), lipid bodies (LD), and cell wall (CW)

Deciphering the alterations in the cell morphology and ultrastructure of microalgal cells is crucial to gain insights into the halotolerance mechanism. Hence, electron microscopy was used to visualize ASW-grown cells and compared to control. The FE-SEM images of the ASW-cultivated microalga cells showed an increase in cell size (~ 12 μm) as compared to control treated cells (~ 4 μm), as depicted in Fig. 2a, b. Furthermore, the TEM micrographs of control cell showed well-organized organelles with discernible chloroplast (Ch), nucleus (N), mitochondria (M), starch granules (S), and lipid bodies (L) (Fig. 2c). On exposure to salinity, the TEM images showed disorganization in the cellular structure with large accumulation of several lipid droplets with a few starch granules, and the chloroplast was collapsed with no distinct demarcation of the thylakoids or stroma (Fig. 2d). The cell wall (CW) of the ASW-cultivated cells was thicker (0.20 ± 0.01 μM) as compared to the control (0.12 ± 0.02 μM) (Fig. 2c, d).

### Effect of salinity on lipids composition and EPS formation

**Fig. 3:**
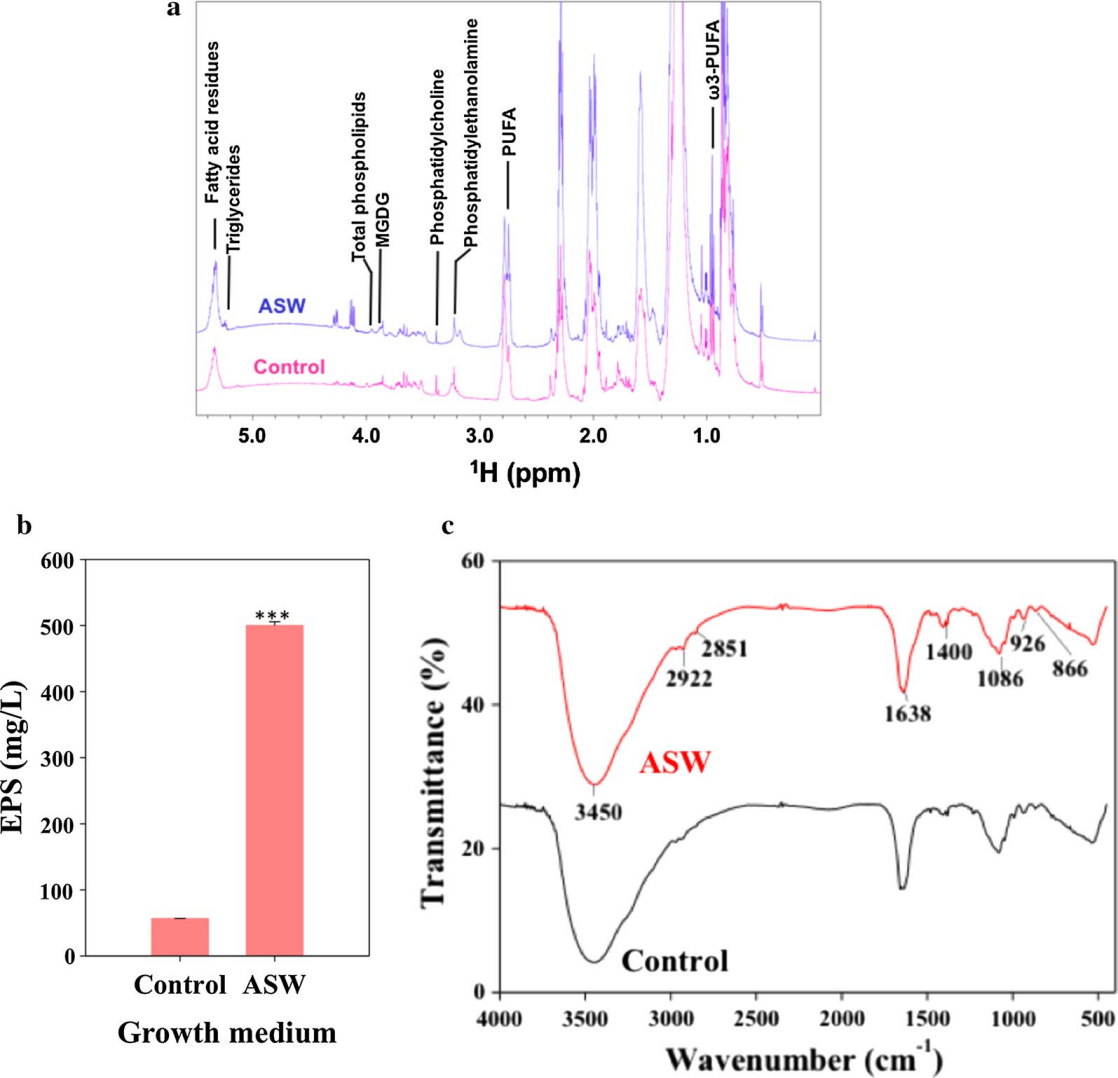
**a** One-dimensional ^1^H NMR spectra of total lipid extracted from control (red) and ASW-cultivated cells (blue). **b** Changes in the EPS production. **c** FT-IR of EPS extracted from control and ASW cultures of *Scenedesmus* sp. IITRIND2 on 7th day

The rearrangement of lipids particularly in membranes is crucial to protect the cells from high salt environments. To this end, we studied the changes in the total lipid composition under halotolerance conditions using ^1^H NMR (Fig. 3a). The ^1^H NMR showed distinct peaks for different classes of lipids including polyunsaturated fatty acids (PUFA), phosphatidylcholine (PC), phosphatidylethanolamine (PE), total phospholipids (PL), triacylglycerides (TAG’s), fatty acid residues, mono galactosyl diacylglycerol (MGDG), and ω^3^ PUFA, respectively (Fig. 3a and Additional file 1: Table S2). Interestingly, all the classes of lipids showed an increase in total lipids extracted from ASW-cultivated cells as compared to control (Fig. 3a). The maximum increase was noted in TAGs (~ 17-fold) followed by the other classes of lipids (Additional file 1: Table S2). These NMR observations corroborate well with the changes in the ultrastructure of *Scenedesmus* sp. IITRIND2.

Parallel to perturbations in the lipid composition, the presence of extrapolysaccharide membrane (EPS) layer around the microalgal cell reduces the penetration of toxic Na^+^ and Cl^−^ ions through the cell membrane which in turn aids its survival under salt stress [36]. *Scenedesmus* sp. IITRIND2 cultivated in ASW showed a 30-fold increase in EPS production as compared to control cells (Fig. 3b). Compositional analysis of the EPS from ASW-cultivated cells showed ~ fourfold increase in total carbohydrates (20 ± 0.25 mg/mL) as compared to control cells (5.2 ± 0.03 mg/mL), while negligible amount of protein was detected in both the samples. Furthermore, to obtain structural information, we analyzed the EPS using FT-IR spectroscopy (Fig. 3c). The spectra showed clear transmittance at 3500–3300 cm^−1^ indicating O–H stretching and 2915–2935 cm^−1^ of asymmetric C–H stretching which is common to all the polysaccharides, while peak from 1000 to 1200 cm^−1^ corresponds to the presence of carbohydrates [37, 38]. The peak around 1640 cm^−1^ indicated the presence of mannose/galactose and peak at ~ 1400 cm^−1^ corresponded to galacturonic acid [36, 38, 39]. The characteristics peak at ~ 860 cm^−1^ showed the presence of α-configuration glucose and peak at ~ 890–900 cm^−1^ indicated the presence of β-d-glucans [36]. Furthermore, less pronounced peaks ~ 1536 cm^−1^ indicated the absence of proteins in the EPS samples.

The compositional and structural analysis presented in the above sections point towards the fact that *Scenedesmus* sp. IITRIND2 has most probably rewired its metabolic pathways to tolerate the salinity stress and hence forth to accumulate high lipid content. Thus, to unravel its molecular responses, we have adapted an integrated omics approach comprising of metabolomics, proteomics, and RT-PCR analysis as discussed in the sections below.

### Changes in the metabolite composition of *Scenedesmus* sp. IITRIND2 in response to salinity stress

**Fig. 4:**
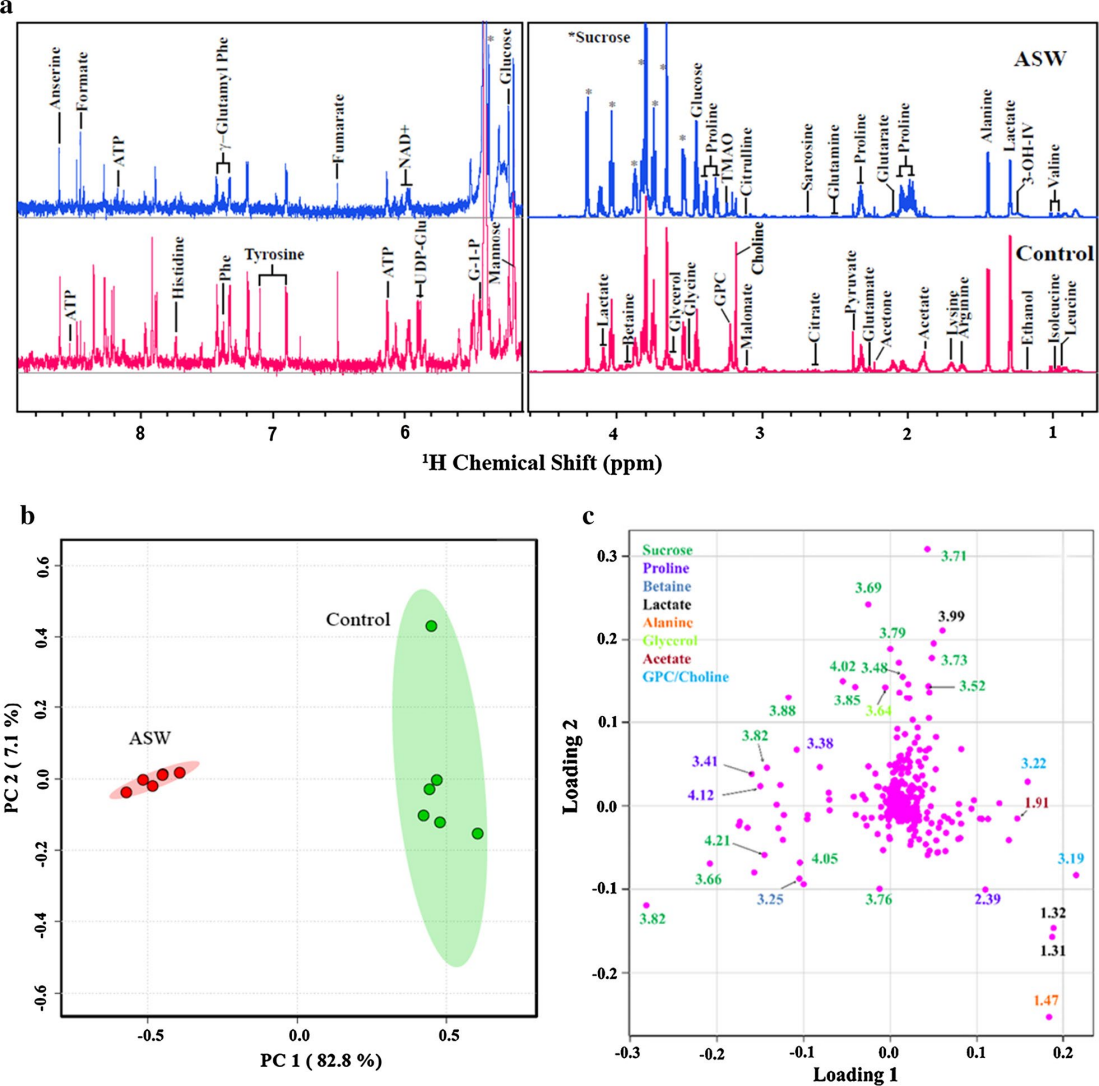
**a** Cumulative 1D ^1^H NMR spectra (*n* = 6) of *Scenedesmus* sp. IITRIND2 Control polar extracts (pink) stacked up with that of ASW cultures (blue). The spectral peaks were assigned for particular small-molecule metabolites. The water region at *δ* 4.6–4.9 was removed for clarity. The abbreviations used are: 3-OH-IV: 3-hydroxyisovalerate; Gln: glutamine; Glu: glutamate; EA: ethanolamine; Cys: cysteine; Cho: choline; PC: phosphocholine; GPC: glycerophosphocholine; DSS: 4,4-dimethyl-4-silapentane-1-sulfonic acid; TMAO: trimethylamine-*N*-oxide; DMG: *N*,*N*-dimethylglycine; Phe: phenylalanine; ATP: adenosine triphosphate; UDP glucose: uridine diphosphate glucose. **b** Combined PCA 2D score plot resulted from the analysis of 1D ^1^H CPMG spectra of *Scenedesmus* sp. IITRIND2 cultivated in control and ASW medium (green = control; red = salt). The semi-transparent red and green ovals represent the 95% confidence interval. **c** PCA loadings plot revealing the metabolites of responsible for the discrimination pattern, the more the metabolite is away from the origin (0,0) more it contributes in the group discrimination

In addition to the changes in the membrane permeability, halotolerant microalgae accumulate low-molecular-weight compatible solutes termed as metabolites which aid in osmoregulation, homeostatic balance, and in maintaining the membrane and protein integrity, etc. [40]. The metabolite profile of *Scenedesmus* sp. IITRIND2 cultivated in ASW as compared to control was analyzed using ^1^H NMR spectroscopy (Fig. 4a–c). The cumulative ^1^H NMR spectra of ASW-cultivated microalgal cells identified a total of 44 metabolites including amino acids (15), organic acids (09), sugars (05), phosphagen (02), osmolytes (03), nucleotides (03), and others (07), respectively (Additional file 1: Table S3). The intensity profiles of ^1^H NMR resonances showed a clear metabolite difference between control and ASW-cultured cells (Fig. 4a). To statistically validate these differences across the two groups, multivariate analysis was performed first using principal component analysis (PCA) (Fig. 4b). The PCA analysis showed clear clustering of the six biological replicates and distinct difference between the control and ASW cultures (Fig. 4a). The PCA loading plot also revealed the metabolites responsible for the observed discrimination pattern (Fig. 4c). Data obtained from the univariate analysis were represented using hierarchically clustered heat maps which showed quantitative data of significantly altered metabolites between the two experimental groups (Additional file 1: Fig. S1).

Synthesis of osmolytes is one of the criteria for identifying different halotolerant microalgal species [41]. For example, based on the accumulation of imino acid, proline, four distinct groups have been categorized including: (1) high proline accumulation as the major osmolyte, e.g., marine microalga; *Chlorella autotrophica*; (2) high proline accumulation along with large quantity of other osmotic solutes such as sorbitol (e.g., *Stichococcus bacillaris*) or asparagine (e.g., *Agrostis stolonifera*); (3) low proline synthesis but accumulate other organic solutes in high amounts such as glycerol (e.g., *Dunaliella* sp.), and (4) low proline accumulation along with low capacity to synthesize the other osmolytes [41, 42]. Based on the metabolomics results, *Scenedesmus* sp. IITRIND2 can be categorized under group 2 as high accumulation of proline along with sucrose was observed (Additional file 1: Table S3). Such high accumulation of proline and sucrose has been reported in various algae including *Synechocystis*, *Microcoleus vaginatus Gom*., *Chlorella autotrophica*, *Stichococcus bacillaris*, *Picochlorum,* and *Neochloris oleoabundans* [19, 40, 41, 43].

### Assessing the differential proteome response of *Scenedesmus* sp. IITRIND2 using MALDI–TOF–MS/MS

**Fig. 5:**
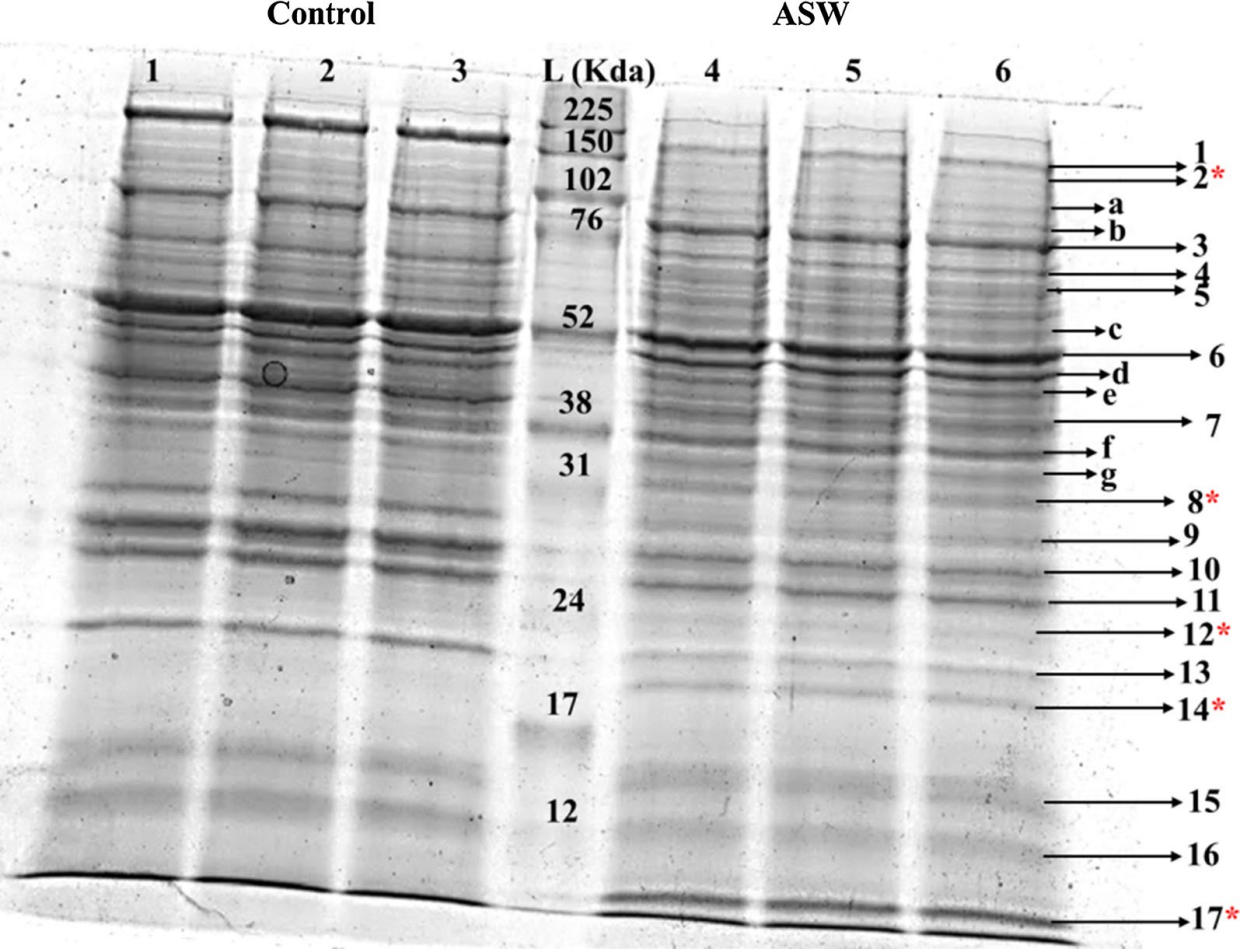
1D SDS PAGE gel showing various protein bands. Lanes 1–3 and 4–6 represent the three replicates of control and ASW samples, respectively. Lane L is protein ladder. Bands 1–17 are differential expressed proteins with a Mascot score > 55; bands a–g are differential expressed proteins with a Mascot score < 55; and * denotes the proteins that are only expressed in ASW cultures

Parallel to the perturbations in the metabolite levels, reorganization of gene expression (reflected in the proteome) of the halotolerant microalga is essential for adaptation to the altered physiological state in response to salinity stress. To this end, proteomics was applied at the early stationary phase (7th day), to reveal the differential expression of proteins in response to salinity stress. 1D SDS PAGE was used to excise 24 unique protein bands, and also to identify the differentially expressed proteins based on their presence/absence, up regulation/down regulation, that are subsequently identified using MALDI–TOF–MS/MS against the Uniprot Chlorophyta database (Fig. 5a). Among the excised bands, only 17 bands showed mascot score above 55, and thus, only these were included in the result and discussion of the present study (Table 1).

**Table 1:** List of identified 1D-resolved protein spots from control and ASW responsible in *Scenedesmus* sp. IITRIND2

Band ID	Identification in	Protein name	Function categories	Theoretical/actual MW (KD)	Mascot score	Coverage (%)	Subcellular location	Fold change
1	A0A1D1ZZ74_AUXPR	Uncharacterized protein	Unknown	200/207.9	57	14	Unknown	3.5 (↓)
2	C1E424_MICC	Uncharacterized protein (fragment)	Helicase activity (predicted)	150/111.5	88	14	Unknown	–
3	K8F2R1_9CHLO	Malic enzyme	Lipid biosynthesis	76/78	69	15	Mitochondria	1.4 (↑)
	A0A087SEW0_AUXPR	Alpha-1,4 glucan phosphorylase	Starch synthesis	76/100.2	77	19	Chloroplast	
4	C1DY39_MICCC	Peptidylprolyl isomerase	Protein degradation	70/63.3	76	13	Cytoplasm	4.3 (↑)
	A0A0D2LKL19_CHLO	Glucose-6-phosphate isomerase	Glycolysis	70/64.59	144	27	Cytoplasm	
5	Q6PYY4_OSTTA	Starch synthase	Starch synthesis	60/58.7	74	16	Chloroplast	1.78 (↓)
	E1ZTI5_CHLVA	Glucose-1-phosphate adenylyltransferase	Starch synthesis	60/55.6	75	19	Chloroplast	
6	A0A172C330_9CHLO	Ribulose bisphosphate carboxylase large chain (fragment)	Calvin cycle	52/36.59	69	30	Chloroplast	1.34 (↓)
7	C0SKA4_9CHLO	Tubulin beta chain (fragment)	Flagellar motion/cytoskeleton microtubule	40/41.6	87	22	Cytoplasm	1.42 (↓)
8	A8JHU0_CHLRE	Malate dehydrogenase	TCA cycle	31/38.8	59	16	Mitochondria	–
	A0A059LS29_9CHLO	Catalase	Antioxidant	31/57.37	96	13	Cytoplasm	
	A0A0D2M0Q2_9CHLO	3-ketoacyl-CoA synthase	Lipid biosynthesis	31/33.9	71	21	Chloroplast	
9	A0A097KLR8_9CHLO	Mg-protoporphyrin IX chelatase	Chlorophyll synthesis	27/39.6	77	36	Chloroplast	1.5 (↓)
10	A0A150H154_GONPE	Uncharacterized protein	Unknown	26/31.4	71	21	Unknown	1.2 (↓)
11	A0A1D1ZTJ7_AUXPR	Uncharacterized protein	Unknown	25/20.87	68	20	Unknown	1.1 (↓)
12	D8TLP0_VOLCA	Glutathione peroxidase	Detoxification of H_2_O_2_	24/22.27	67	11	Cytoplasm	–
13	A0A061S0Z1_9CHLO	Phosphodiesterase	Hydrolase activity	20/48.2	52	14	Cytoplasm	2.3 (↓)
14	A8J7H6_CHLRE	Thioredoxin-like protein	Antioxidant	19/27.6	67	29	Cytoplasm	–
	A0A06IRYCR_9CHLO	Pyrroline-5-carboxylate reductase	Proline synthesis	19/28.3	78	29	Cytoplasm	
	A0A1C9ZQC69_CHLO	Alfin-like protein	Salt tolerance	19/20.1	52	17	Cytoplasm	
15	C1KR5_MICCC	50S ribosomal protein L14	Protein synthesis	15/13.40	87	40	Chloroplast	1.1 (↑)
	Q946N2_9CHLO	Ribulose 1,5-bisphosphate carboxylase/oxygenase large subunit (fragment)	Calvin cycle	15/21.8	71	45	Chloroplast	
16	A0A06IQQ83_9CHLO	Nadh:ubiquinone oxidoreductase 13 kDa subunit	Oxidative phosphorylation	12/16.3	52	20	Chloroplast	1.2 (↓)
17	A0A061QN19_9CHLO	Putative salt tolerance-like protein (fragment)	Salt tolerance	10/7.9	54	48	Unknown	–

For visualization interpretation, single (↑, ↓) arrows are used to represent relative change in the value of protein expression (as evaluated from their intensities using the Image J software

The identified proteins were functionally grouped into 09 different biosynthesis categories using QuickGO, gene annotation, and ontology tool including lipid biosynthesis (02), starch metabolism (02), photosynthesis (03), glycolysis and TCA cycle (03), detoxification and antioxidants (04), salt tolerance (02), protein metabolism (03), cellular structure (01), and unknown (04), respectively (Table 1). The characteristic details of identified proteins including the accession no (Uniprot database), protein name, functional category, theoretical molecular weight, Mascot score, coverage, and subcellular location are summarized in Table 1. Furthermore, on the basis of protein annotation, similarity search for *Scenedesmus* sp. IITRIND2 against freshwater and marine microalgal species was done to shed light on the phylogenetic relationship (Additional file 1: Fig. S2). The results revealed that fresh water species, *Desmodesmus communis* (12%), followed by *Chlamydomonas reinhardtii* (11%) and marine algae *Ostreococcus tauri* (8.33%) showed maximum similarity with the proteome of *Scenedesmus* sp. IITRIND2, thus indicating its proximity to both marine and fresh water species. Such unique distribution of proteome can also potentially contribute to the novel halotolerant nature of this fresh water green alga.

### Gene-expression analysis of major metabolic pathways using RT-PCR

**Fig. 6:**
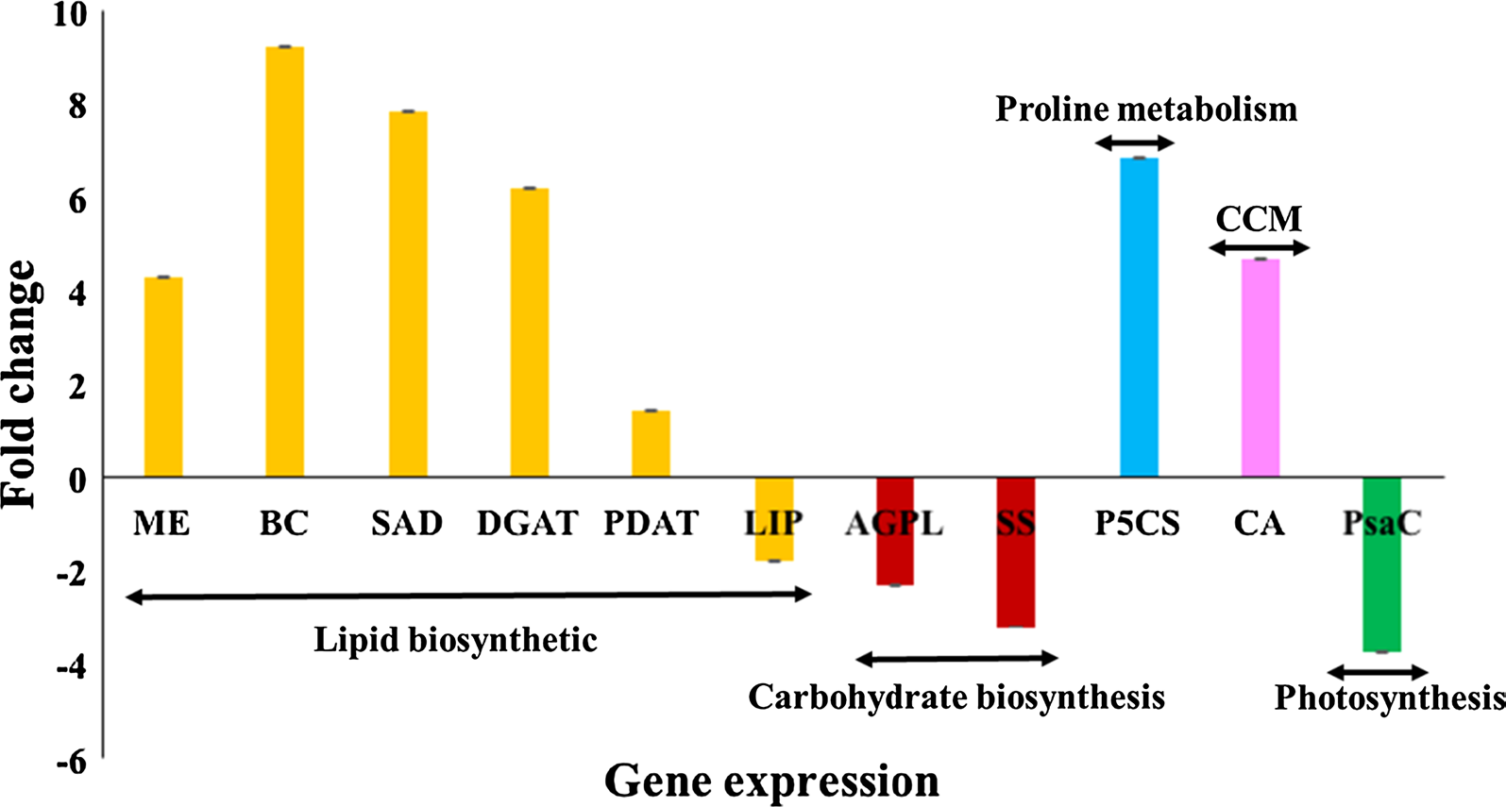
Fold change of genes expression as analyzed by RT-PCR

Substantiation of differential protein expression profiles using gene-expression analysis will delineate the metabolic pathways that are crucial for halotolerance behavior of *Scenedesmus* sp. IITRIND2. To analyze the relative gene-expression patterns, six lipid biosynthesis, two carbohydrate synthesis genes, carbon concentration mechanism (CCM), proline biosynthesis, and photosynthesis were studied. Compared to the control, all the lipid biosynthetic genes showed an increase in ASW except LIP. Among the elevated genes, lipid biosynthesis showed maximum expression (BC > SAD > DGAT > ME > PDAT) (Fig. 6). On the other hand, both carbohydrate synthesis genes expression decreased by two-to-threefold in ASW as compared to control cells. Furthermore, a five-to-sevenfold increase in proline and CCM genes expression was recorded in ASW-cultivated cells with down regulation of photosynthesis gene as compared to control cells (Fig. 6).

## Discussion

### Salinity-driven physiological and molecular responses of high lipid accumulating alga *Scenedesmus* sp. IITRIND2

Integrated omics approach studies are helpful in facilitating the correlation of metabolic pathways by linking gene, proteins, and metabolites of any organism [44]. In the last decade, researchers have made a progress in unraveling the microalgae complex biosynthetic pathways by utilizing various omics techniques [18, 44]. These algal-omics studies have led to well-established metabolic maps which aided the identification of targets for genetic engineering to enhance lipid accumulating ability of microalgal strains [45, 46]. In the present study, an “integrated algal-omics approach” was adopted to unfold the halotolerance mechanism of a novel high lipid accumulating microalgal strain *Scenedesmus* sp. IITRIND2, and also identified potential genome editing targets.

**Fig. 7:**
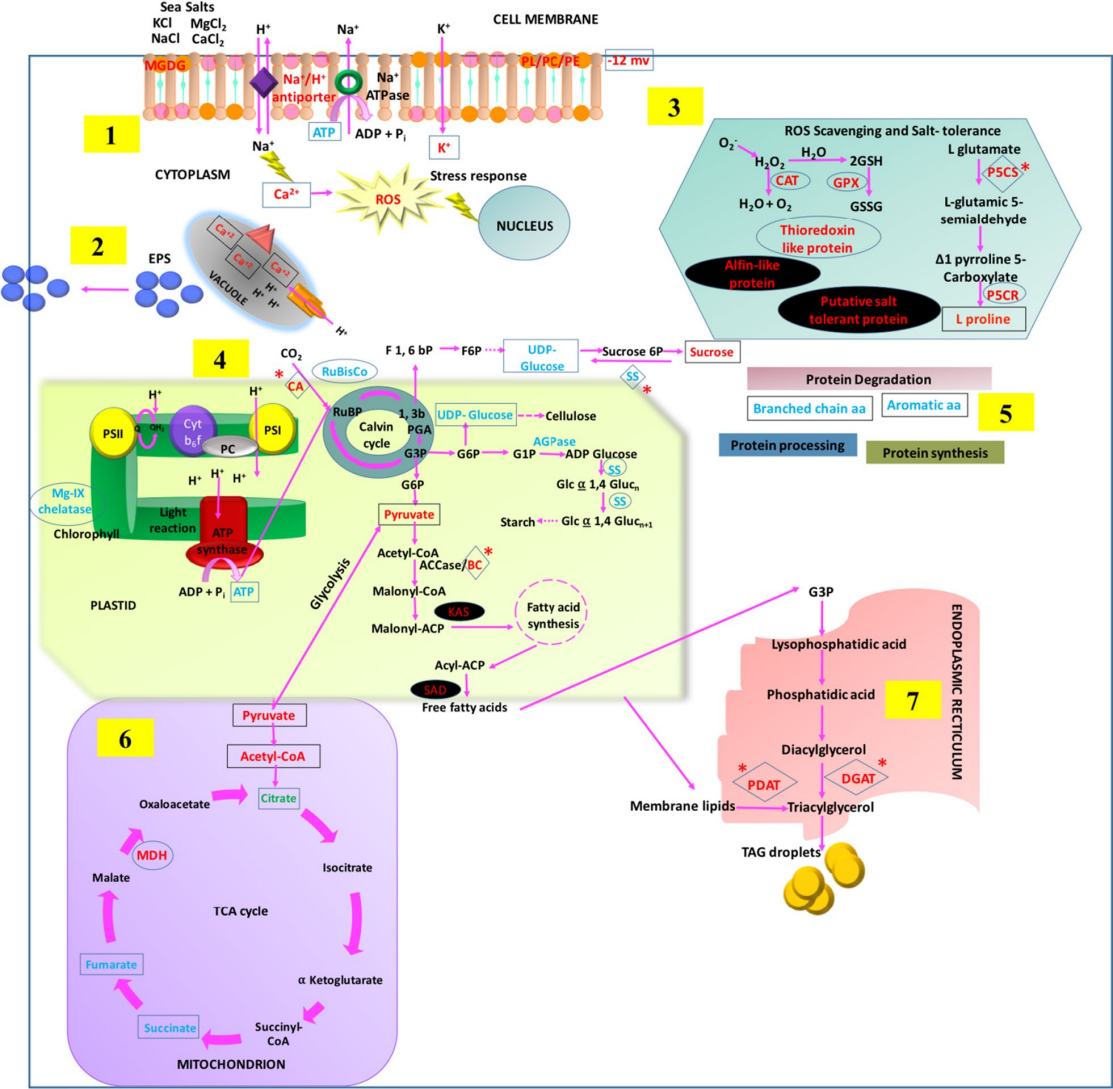
Schematic representation of salt-tolerant mechanism deployed by *Scenedesmus* sp. IITRIND2 by alteration in various cell organelles/pathways: (**1**) ion channels and cell membrane, (**2**) production of EPS, (**3**) ROS scavenging and salt tolerance, (**4**) lipid and carbohydrate metabolism and photosynthesis, (**5**) protein metabolism, (**6**) TCA cycle, and (**7**) TAG synthesis. The significant genes are outlined with diamonds and *; proteins with circles and metabolites/ions with squares. The up regulated genes/proteins/metabolites are shown in red, while in blue for down regulation. The four potential biomarkers for genetic engineering are highlighted in black background

To survive the salinity stress, *Scenedesmus* sp. IITRIND2 essentially needs to maintain the osmotic equilibrium across the membrane and cytoplasm which was achieved via alterations in the membrane permeability, cell surface charge, and lipid composition along with perturbations in the gene, protein, and metabolites biosynthesis. Based on the results obtained from biochemical, structural, and integrated algal-omics studies, we propose a salinity-driven metabolic pathway of the microalga, which enabled it to tolerate the salt stress along with high lipid accumulation (Fig. 7).

Initially, under salinity stress conditions, the microalga adapted by changing the membrane potential and permeability, as an increase of the K^+^ ions inside the microalga indicated it to be an important coping mechanism which help to reduce the toxic effects of enhanced Na^+^ ions (Fig. 7: panel 1) [47]. An increase in uptake of Ca^2+^ also helps the microalgae to counteract the toxic effects of Na^+^ ions, and thus reduce the permeability of the plasma membrane [48]. Parallel to the ions balance, fluctuations in the lipid composition particularly the membrane lipids was also an essential acclimatization mechanism deployed by *Scenedesmus* sp. IITRIND2. The ^1^H NMR showed increase in the negatively charged lipids including PE, PC, and PL which contributed towards the negative charge of the cell membrane under halotolerant conditions as substantiated by the decrease in its surface zeta potential (Figs. 3a, 7: panel 1). Such an increase in the negatively charged lipids has shown to shield the microalgal cell membrane from the Na^+^ ions and avoid membrane destabilization [49]. Similar results have been reported in various microalgae including *D. salina* and *Chlamydomonas nivalis* [50, 51].

An increase in the saturated fatty acids (SFA’s) in the algal plasma membrane is crucial to decrease its permeability towards the small ions (Na^+^, Cl^−^) but, at the same time, maintains it fluid and flexible enough to carry out normal cellular processes [35, 52]. Earlier, we reported a twofold increase in the monounsaturated fatty acids (MUFA) content particularly of C18:1 followed by a 1.5-fold increase in C16:0 in the fatty acid composition under salinity stress [9]. An increase in SFA/MUFA’s content such as C16:0 and C18:1 in microalgae helps in maintaining the fluidity of cell membrane; as highly unsaturated, short, and branched fatty acids will reduce the fluidity and permeability of the cell membrane, thereby restricting the osmolytes balance [47–49]. Furthermore, a twofold increase was observed in the MGDG levels (Fig. 3a), which is a non-bilayer forming lipid that balances the membrane fluidity, and is also crucial for photosynthetic processes involving lateral, rotational, and transmembrane diffusion during electron transfer from photosystem II (PSII) and photosystem 1 (PSI) [53]. The cells cultivated in ASW also showed an increase in EPS formation which can further enhance its salinity tolerance characteristics (Figs. 3b, 7: panel 2).

Apart from the modulation of the membrane permeability, increase in the accumulation of osmolytes was observed to an important phenomenon for restoring the osmotic equilibrium (Additional file 1: Table S3 and Fig. 7: panel 3). High concentrations of proline (tenfold) and sucrose (sixfold) in ASW-cultivated *Scenedesmus* cells suggested that these two be the major osmolytes engaged in regulating the internal osmotic balance (Additional file 1: Table S3 and Fig. 7: panel 3, 4). Proline is actively involved in the restoration of turgor pressure, regulation of cellular water structure, reactive oxygen species scavenging (ROS), inhibition of lipid peroxidation, and as an antioxidant [54]. On analysis of the proteomics of the ASW cells, unique band was observed of pyrroline-5-carboxylate reductase (P5CR) (Table 1). l-Proline is synthesized from l-glutamate via two successive reactions catalyzed by the P5CS and P5CR, with consumption of one molecule of ATP and two molecules of NADPH [55]. Indeed, RT-PCR analysis showed the overexpression of P5CS, thus substantiating the metabolic and proteomic results related to l-proline increase in ASW cells (Fig. 6). A significant reduction in the levels of glutamate and ATP also indicated their flux towards proline synthesis (Additional file 1: Table S3). Apart from proline accumulation, sugar accumulation also contributes, towards the maintenance of osmotic strength, the microalgal cells under salt stress. Such an accumulation of sugars not only prevents cellular degradation but also provides a source of energy under saline conditions [13]. Among the sugars, the omics data indicated that sucrose was synthesized prior to other sugars as an apparent decline in the level of UDP glucose and down regulation of sucrose synthase (catalyzes the breakdown of sucrose) was recorded (Additional file 1: Table S3 and Figs. 6, 7: panel 4). Sucrose plays a vital role in decreasing the toxicity of Na^+^ ions by aiding its active extrusion, inhibition of membrane vesicles, and improving the H^+^-ATPase activity [43]. The metabolomics also showed a rise in the levels of glucose and mannose/trehalose in the ASW-cultivated cells. Trehalose has an established role in stabilization of proteins under salt stress by acting as an antioxidant and osmoregulatory molecule [56]. Another important adaptive mechanism involves the quenching of the ROS generated due to salt stress. *Scenedesmus* sp. IITRIND2 efficiently grew in ASW due to differential expression of three antioxidant proteins including thioredoxin like protein, glutathione peroxidase, and catalase (Table 1 and Fig. 7: panel 3). Interestingly, *Scenedesmus* sp. IITRIND2 exhibited distinct salt-tolerant response as proteomics data identified two unique proteins: (a) putative salt-tolerant protein and (b) Alfin-like protein (Table 1 and Fig. 7: panel 3).

Excessive intrusion of Na^+^ ions into the cells due to salt stress can inhibit the amino acid/protein biosynthesis. A decrease (~ 1.2 fold) was observed in the levels of all the aromatic and branched chain amino acids under salt stress (Additional file 1: Table S3). The salt-induced stress on protein folding machinery is also evident as the halotolerant cells showed for up regulation of peptidylprolyl isomerase (PPI), a potential molecular chaperon and ROS scavenger (Table 1 and Fig. 7: panel 5) [57]. As chloroplast is one of the major sources of ROS production, suppression of photosynthesis to combat for oxidative damage under salt stress. A reduction in the expression of Mg-protoporphyrin IX chelatase, ribulose bisphosphate carboxylase large chain (fragment), and PsaC in ASW cells indicated suppression pf photosynthesis (Table 1 and Figs. 6, 7: panel 4). Furthermore, as salinity decreases the solubility of the CO_2_ in water, thereby decreasing its availability to the microalgal cells, an increase in the gene-expression levels of carbonic anhydrase (CA) was recorded (Figs. 6, 7: panel 4). It has been reported that, under stress conditions, the levels of CA increases which facilitates CO_2_ acquisition along with the activation of C4 CCM [58, 59].

A subsequent up regulation of malate dehydrogenase catalyses the conversion of malate to oxaloacetate in TCA cycle in ASW cultures which indicated accumulation of acetyl-CoA: precursor for fatty acid synthesis (Additional file 1: Table S3, Table 1 and Fig. 7; panel 6). An up regulation in the levels of malate dehydrogenase has been related to salt adaptation in *Arabidopsis* [60]. A decline in the starch metabolism was recorded as proteomics/RT-PCR analysis of ASW cultures which showed down regulation of starch synthase and glucose-1-phosphate adenylyltransferase (AGPase) (Table 1 and Figs. 6, 7: panel 4). In addition, up regulation in the glycolysis (glucose-6-phosphate isomerase and glucose) and down regulation of TCA cycle (succinate and fumarate) indicated redirection of cell’s carbon flux towards lipid body formation (Additional file 1: Table S3 and Table 1). Such a rewiring of metabolic pathway towards synthesis of TAGs is a survival trait adopted by the microalga to survive under stress conditions.

In a recent study, we observed an increase in TAG accumulation (52% of dry cell weight) in microalgal cells cultivated in ASW as compared to control cells which is well corroborated with the TEM micrographs showing deposition of large number of intracellular lipid droplets (Fig. 2c, d) [9]. These results were complimented by the proteomics and gene-expression analysis as lipid biosynthesis genes (ME, BC, KCS, SAD, DGAT, and PDAT) were up regulated in ASW cultures (Table 1 and Figs. 6, 7: panel 2, 6). An apparent up regulation in the levels of ME at both transcriptional (RNA) and translational (protein) levels indicates the elevated conversion of malate to pyruvate, thus leading to the generation of more NADPH and the direction of its flux towards lipid synthesis (Additional file 1: Table S3, Table 1 and Figs. 6, 7: panel 3). Increase in ME has been reported under nitrogen, depletion, CO_2_ deprivation, and salinity stress, respectively [19, 27, 61].

The first committing step towards the fatty acid synthesis is the conversion of acetyl-CoA to malonyl-CoA catalyzed by acetyl-coA carboxylase (ACCase) [62]. BC is the part of ACCase that catalyzes the carboxylation of acetyl carrier protein (ACP) and transfer of carboxyl group, thereby generating malonyl-CoA [63]. On the other hand, KCS catalyzes the conversion of malonyl-coA to acyl-coA, the first rate-limiting step in fatty acid elongation, while SAD is responsible for the conversion of stearoyl-ACP to oleoyl-ACP, and is crucial for maintaining the ratio of saturated fatty acids and unsaturated fatty acids [64, 65]. Augmentation in the levels of DGAT that catalyze the conversion of diacylglycerol (DAG) to TAG was observed. In addition, PDAT which is responsible for the conversion of chloroplastic membrane lipids to TAGs was also enhanced. These observations establish that both de-novo and lipid recycling pathways are essential and responsible for the overall increase in TAG content under salinity stress conditions (Fig. 7: panel 7).

In a nutshell, integrated omics results on *Scenedesmus* sp. IITRIND2 have unraveled four unique genetic targets, whose overexpression could potentially convert fresh water microalgae to high lipid accumulating halotolerant species. These four targets include: (a) Alfin-like protein, (b) putative salt-tolerant protein, (c) KCS, and (d) SAD. Alfin-like protein family was discovered in alfalfa (*Medicago sativa*), and its overexpression in parent plant and *Brassica napa* resulted in increasing the salt tolerance [64, 66]. This is the first report showing the differential expression of Alfin-like protein in an algal strain, and could be a potential genetic engineering target in microalgae for increasing the halotolerance and warrants further investigation. On the other hand, putative salt tolerance protein could be a possible homolog of salt-tolerant protein (STO) identified in *Arabidopsis thaliana* which is an Na^+^/H^+^ antiporter, whose overexpression has resulted in increased salt tolerance [67, 68]. KCS and SAD which are involved in the lipid biosynthesis pathway are the two other important genetic targets revealed from the current study. They together are responsible for MUFAs’ formation, and also help to acclimatize the algal intracellular membrane compartment to function in high internal osmolytes concentration [65].

## Concluding remarks

A novel halotolerant microalga, *Scenedesmus* sp. IITRIND2, modulated its cellular machinery to adapt in high saline environments which was reflected in changes to the ion channels, membrane permeability, ultrastructure, metabolites, and proteome. The observed metabolic rewiring in the microalgae is unique and different from common halotolerant microalga: *Dunaliella salina*. The investigation helped to identify four biomarkers including KCS, SAD, Alfin-like protein, and putative salt-tolerant protein that could be potential genetic engineering targets to generate oleaginous halotolerant species.

The present study also pointed out the ease of harvesting the microalgal biomass cultivated in ASW. The cells showed an increase in cell size, EPS formation, and loss of β tubulin chain expression which is crucial for motility, thereby enhancing the auto-sedimentation property and thus reducing the cost of downstream processing. Another interesting aspect for reducing the cost of algal production is biorefinery approach which integrates biodiesel production with conversion/extraction of all the available compounds into spectrum of marketable high value compounds such as omega 3 fatty acids, bioactive compounds, etc. without the generation of waste [3]. *Scenedesmus* sp. IITRIND2 along with augmented lipid accumulation showed high amounts of proline and sucrose which are industrially relevant products. In summary, the outcomes of this study have not only enhanced our existing molecular-level knowledge of halotolerance and lipid accumulation in response to salt stress but also yielded genetic targets for generating novel halotolerant algal strains.

## Additional file

**Additional file 1.** Additional figures and tables.
