# Supplementary material for: Delineating the molecular responses of a halotolerant microalga using integrated omics approach to identify genetic engineering targets for enhanced TAG production
Source: Biotechnol Biofuels. 2019 Jan 4;12:2. doi: 10.1186/s13068-018-1343-1 (PMC6318984; doi:10.1186/s13068-018-1343-1)
Supplement: Supplementary file 1 — Additional file 1. Additional figures and tables. [file 13068_2018_1343_MOESM1_ESM.pdf]

ADDITIONAL FILE 1

Delineating the molecular responses of a halotolerant microalga using integrated omics approach to identify genetic engineering targets for enhanced TAG production

Neha Arora[1], Poonam Kumari[1#], Amit Kumar[3#], Rashmi Gangwar[1#], Khushboo Gulati[1#], Parul A Pruthi[1], Ramasare Prasad[1], Dinesh Kumar[3]*, Vikas Pruthi[1,2]*, Krishna Mohan Poluri[1,2]*

[1]Department of Biotechnology, [2]Centre for Transportation Systems, Indian Institute of Technology Roorkee, Roorkee -247667, Uttarakhand
[3]Centre of Biomedical Research, SGPGIMS, Lucknow – 226014, Uttar Pradesh, India

***Corresponding Authors**

Dr. Dinesh Kumar

Email: dineshcmbr@gmail.com

Prof. Vikas Pruthi

Email: vikasfbs@iitr.ac.in

Dr. Krishna Mohan Poluri

Email: mohanpmk@gmail.com; krishfbt@iitr.ac.in

Ph: +91-1332-284779

Fax: +91-1332-286151

[#]Authors Contributed Equally

Fig. S1: Heat maps showing z-scores of discriminatory metabolite entities in ASW cells compared to control culture. X-axis represents the replicates of the culture (**A**); (Control – lane (1-6) - red bar (**B**); ASW – lane (7-12) The color scheme through signifies the elevation and reduction in metabolite concentration in ASW compared to control : dark blue: lowest; dark red: highest.

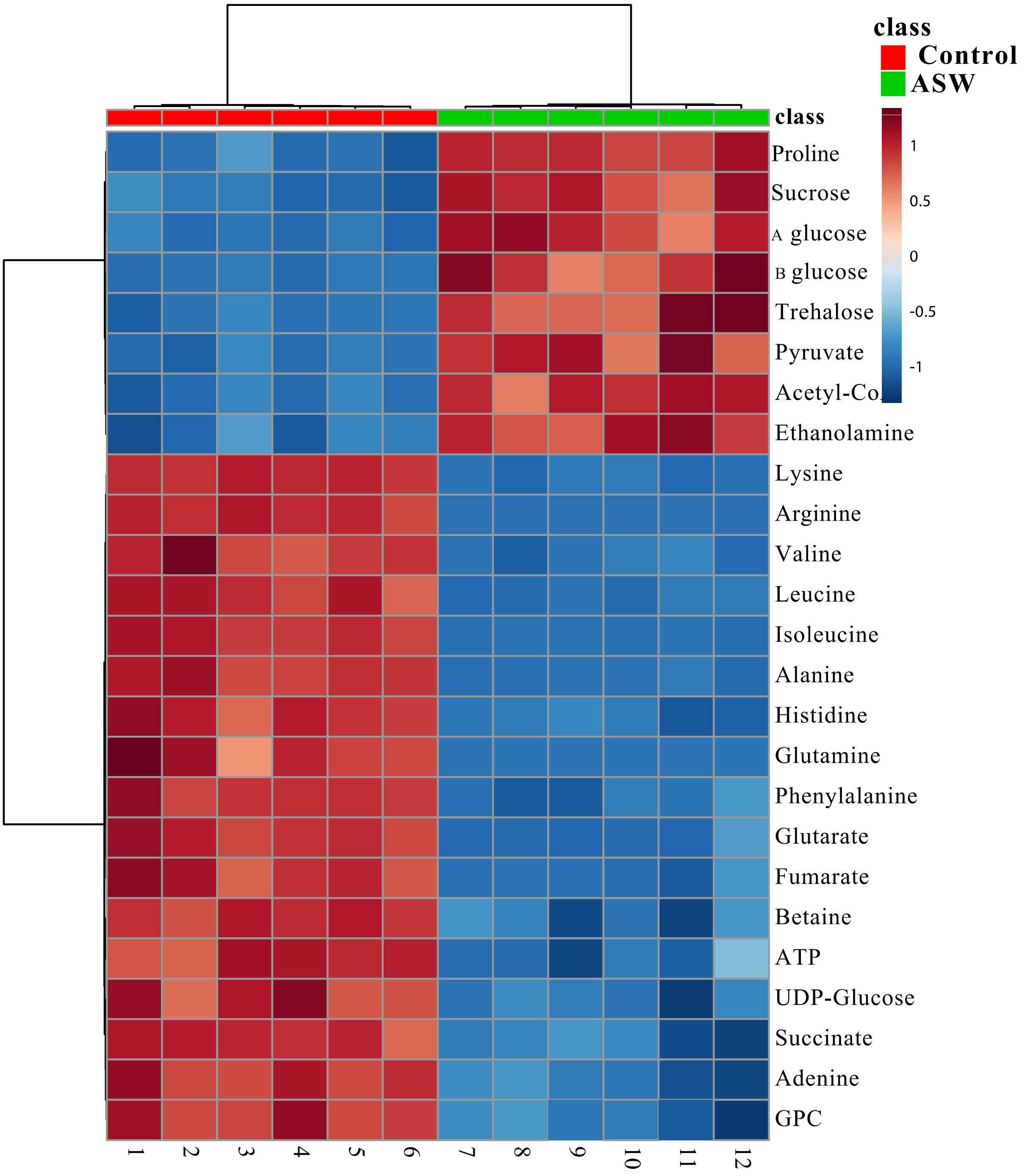

Fig. S2: Similarity search for *Scenedesmus* sp. IITRIND2 against freshwater and marine microalgal species based on the identified proteins using proteomics studies. The functional annotation and grouping of the identified proteins was done by QuickGo, EMBL-EBI (https://www.ebi.ac.uk/QuickGO/) using Explore biology tool. The annotation and proteomics analysis suggested that the identified proteins of *Scenedesmus* sp. IITRIND2 belonged to 15 different algae which comprises of both fresh water and marine species. For fresh water species, the maximum hits were related to *Desmodesmus communis* (12 %), followed by *Chlamydomonas reinhardtii* (11 %) and *Ettlia pseyudoalveolaris* (10 %) respectively. On the other hand, for marine algae, maximum similarity was recorded with *Ostreococcus tauri* (8.33 %) respectively.

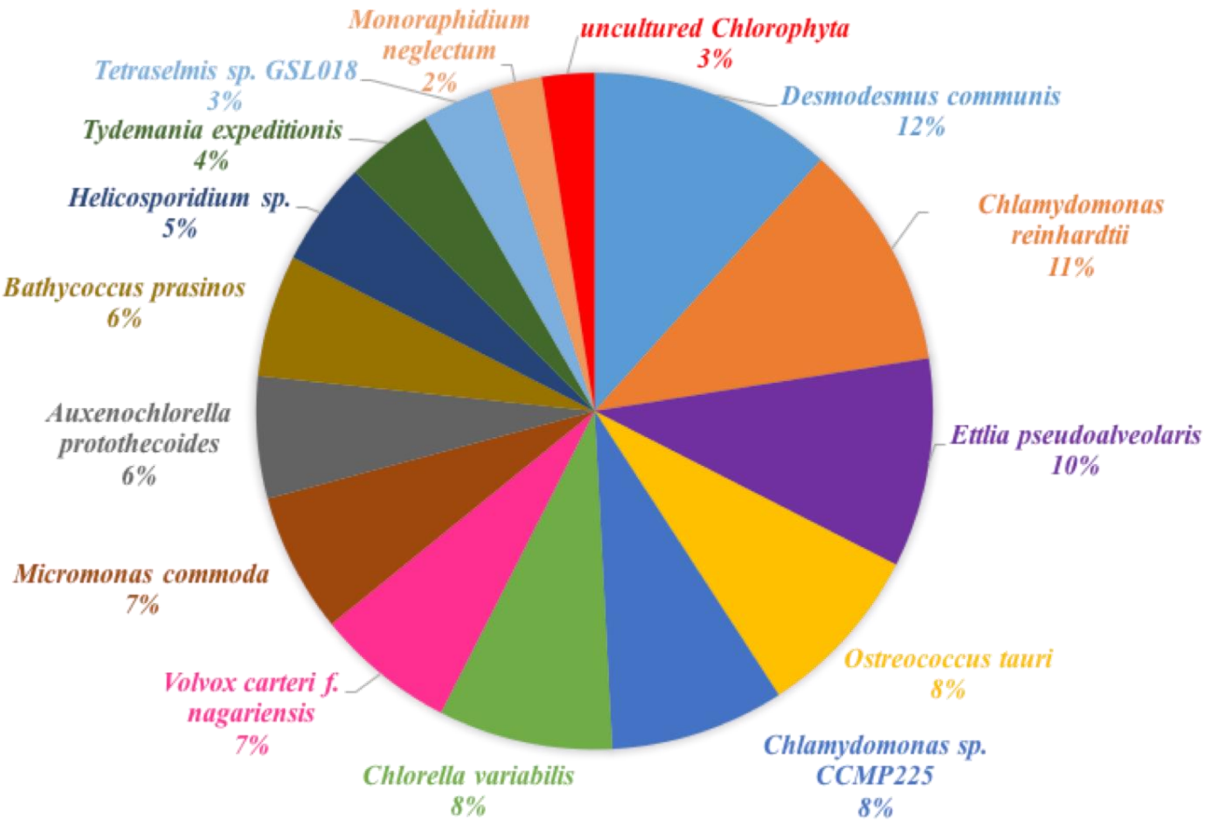

Table S1: List of primers used in the expression analysis (RT-PCR)

Gene	Forward	reverse	Tm	GC	Reference
**Beta Actin**	ACATCAAGGAGAAGCTGGCCT A	ATGTCGACGTCGCACTTCATGA	F 54.8	50	-
			R 54.8	50	
**DGAT-3280**	GGCACAAAGAGTTCACCGT	ACAAACTTGAGGTGGGTG	F 51.1	53	[1]
			R 48	50	
**ME-3137**	CCCTCTCGTTCCCCTTTTATT	AAATGCTGACGCAAGTGTGA	F 52.4	48	
			R 49.7	45	
**P5CS**	GTGCCCATCGGCGTGCTTCT	CGTGTTGCGCTTGATGTGGC	F 57.9	65	[2]
			R 55.9	60	
**BC**	TGCGATTGGGTATGTGGGGGT	ACCAGGACCAGGGCGGAAAT	F 56.3	57	[3]
			R 55.9	60	
**SAD**	TCCAGGAACGTGCCACCAAG	GCGCCCTGTCTTGCCCTCAT	F 55.9	60	
			R 57.9	65	
**PGAT**	GGATAAGAGCGGCACAAGGA	GAAGGGCGAGATTGGAATGA	F 53.8	55	[4]
			R 51.8	50	
**LIP**	GGCCTCAAAGCCACCAGTAC	GGCAGTGCACATGTTGCAG	F 55.9	60	
			R 53.2	58	
**SS**	CAGGCAAGGATACATCTACTG	TACTGCCCAACCATCTCATC	F 55.5	47.6	-
			R 56.9	50	
**AGP-L**	CCATGAGCAACTGCATCAAC	GGTTGAGCGAGGTGGAGTT	F 51.8	50	[5]
			R 53.2	58	
**Psac**	GAACATCACCACCACCAGGA	CGGTGCTTGGCTTTTAGTTTG	F 53.8	55	[6]
			R 52.4	48	
**CA**	TGAAGGAGGGCTCTGATGAT	GTTTGCGAATGAGATGGTGT	F 51.8	50	[7]

Table S2: List of lipids along with their respective chemical shifts and fold change as evaluated using Bruker Top spin 3.5 (* indicates the chemical shift taken for fold change estimation).

S.No.	Lipids	Chemical shift (ppm)	Fold change
1	PUFA	2.76 (d)	8.7
2	Phosphatidylcholine	3.38 (s)	3.05
3	Phosphatidylethanolamine	3.22 (s)	4.09
4	Total phospholipids	3.95 (s)	8.13
5	Triglycerides	4.12 (q)*, 4.27 (q), 5.25 (s)	17.2
6	Fatty acid residues	5.32 (m)	10.05
7	MGDG	3.88 (s)	1.9
8	Omega3 PUFA	0.85 (t)*, 0.95 (t)	6.03

Table S3: List of metabolites along with their respective chemical shifts and metabolic fold change patterns as a consequence salt stress. One-way ANOVA followed by post-hoc Tukey's HSD was conducted to determine significant ($p < 0.001$) metabolic changes.

**Metabolite Name**	**Assignment**	**Chemical shifts (δ) in ppm**	**Fold change in treated cells**
		**Amino acids**	
Leucine	δ-CH3<br>δ-CH3	0.95 (d)<br>0.96 (d) [€]	-1.2
Isoleucine	γ-CH3<br>δ-CH3	0.93 (t) [€]<br>1.00 (d)	-1.3
Valine	γ-CH3<br>γ-CH3	0.98 (d)<br>1.03 (d) [€]	-1.3
Arginine	γ-CH2	1.68 (m)[€]	-2.5
Lysine	δ-CH2	1.69 (m)[€]	-1.3
Alanine	β-CH_3<br>α-CH	1.47 (d) [€]<br>3.79 (q)	-1.5
Proline	γ-CH2<br>γ-CH2<br>½ β-CH2<br>½ β-CH2<br>½ β-CH2	1.99 (m)<br>2.00 (m)<br>2.06 (m)<br>3.32 (m) [€]<br>3.41 (dt)	+10.2
Histidine	C4H-ring	7.71 (d)	-1.2
Glutamate	β-CH_2<br>γ-CH_2	2.11 (m) [€]<br>2.34 (m)	-1.1
Glutamine	β-CH_2<br>γ-CH_2	2.12 (q)<br>2.44 (m) [€]	-1.1
Sarcosine[#]	N-CH_3	2.71 (s)	+1.4
Glycine	α-CH_2	3.56 (s)	-
Tyrosine	C2H & C6H<br>C3H & C5H	6.88 (d) [€]<br>7.18 (d)	-
Phenylalanine	C2H & C6H<br>C4H<br>C3H & C5H	7.31 (m)<br>7.37 (m)<br>7.43 (m) [€]	-1.1
γ -glutamyl-phenylalanine	C2H, C6H & C4H<br>C3H & C5H	7.3 (m) [€]<br>7.4 (m)	-
		**Organic acids**	
Lactate	*β*-CH_3<br>*α*-CH	1.32 (d) [€]<br>4.10 (m)	-1.4
Acetate	CH_3	1.91 (s)	-1.5
Aspartate	C6H, C6H	2.8 (m) [€], 2.66 (q)	-1.6
Pyruvate	γ -CH 3	2.4 (s)	+1.5
Glutarate	β, δ- CH2	2.16 (t)	-1.2
Succinate	α, β-CH2	2.39 (s)	-1.9
Citrate	½ γ-CH2<br>½ γ-CH2	2.52 (d) [€]<br>2.69 (d)	-
Fumarate	CH	6.51 (s)	-1.6
Formate	CH	8.44 (s)	-
		**Carbohydrates/sugar**	
Sucrose	C10H<br>C12H<br>C13H	3.46 (t) [€]<br>3.55 (dd)<br>3.66 (s)	+6.1

	C11H<br>C17H & C19H<br>C5H & C9H<br>C4H<br>C3H<br>C7H	3.75 (m)<br>3.77 (m)<br>3.81 (dd)<br>4.04 (t)<br>4.21(d)<br>5.40(d)	
α-Glucose	C4H	3.39 (m)	+3.8
β-Glucose	C5H<br>C3H<br>C1H	3.45 (m)<br>3.47 (m) €<br>5.22 (d)	+2.6
Mannose/Trehalose	C1H	5.19 (d)	+1.9
Glucose-1-phosphate	C1H	5.55 (d,d)	-
**Phosphagen**			
Choline/PC	N–$(CH_3)_3$	3.20 (s)	-2.3
GPC	N–$(CH_3)_3$	3.22 (s)	-1.6
**Osmolytes**			
Glycerol	½ γ-CH2<br>½ γ-CH2	3.63 (d) €<br>3.65 (d)	-
Betaine	N–$(CH_3)_3$<br>β-CH_2	3.26(s) €<br>3.91 (s)	-
TMAO	N–$(CH_3)_3$	3.27(s) €	+3.2
**Nucleotides**			
Adenine	C2H<br>C6H	8.19 (s)	-2.6
ATP	C7H<br>C12H<br>C2H	8.61 (s) €<br>8.25 (s)<br>6.13 (d)	-1.2
NAD+	C28H	6.08 (m)	-1.3
**Others**			
Ethanol	CH3	1.17 (t)	-
Acetyl choline	S-$(CH_2)_2$	3.2 (s)	+1.6
3-hydroxy-isovalerate	γ-CH3	1.24 (s)	-
Acetone	CH_3	2.22 (s)	-
UDP-Glucose	C22H	6 (m)	-2.1
Ethanolamine	N-CH_2	3.13(m)	+2.2
Methanol	CH_3	3.34 (s)	-

Note: All the values of the metabolites were statistically significant with p value 0.001 respectively. "€" represents he metabolite peak used for evaluating the quantitative difference as represented here fold changes in the case of multiple signals/chemical shift values.

References

1. Fan J, Xu H, Luo Y, Wan M. Impacts of CO_2 concentration on growth , lipid accumulation , and carbon-concentrating-mechanism-related gene expression in oleaginous Chlorella. 2015;2451–62.
2. Krell A, Funck D, Plettner I, John U, Dieckmann G. Regulation of proline metabolism under salt stress in the psychrophilic diatom Fragilariopsis cylindrus (Bacillariophyceae). J Phycol. 2007;43:753–62.
3. Liu J, Hua W, Zhan G, Wei F, Wang X, Liu G, et al. Plant Physiology and Biochemistry Increasing seed mass and oil content in transgenic Arabidopsis by the overexpression of wri1 -like gene from Brassica napus. Plant Physiol Biochem. 2010;48:9–15.
4. Jia J, Han D, Gerken HG, Li Y, Sommerfeld M, Hu Q, et al. Molecular mechanisms for photosynthetic carbon partitioning into storage neutral lipids in Nannochloropsis oceanica under nitrogen-depletion conditions. Algal Res; 2015;7:66–77.
5. Li T, Gargouri M, Feng J, Park J, Gao D, Miao C, et al. Bioresource Technology Regulation of starch and lipid accumulation in a microalga Chlorella sorokiniana. Bioresour Technol; 2015;180:250–7.
6. Qian H, Sheng GD, Liu W, Yingcong L, Liu Z, Zhengwei F. Inhibitory effects of atrazine on Chlorella vulgaris as assessed by real-time polymerase chain reaction. Environ Toxicol Chem. 2008;27:182–7.
7. Liu W, Ming Y, Li P, Huang Z. Inhibitory effects of hypo-osmotic stress on extracellular carbonic anhydrase and photosynthetic efficiency of green alga Dunaliella salina possibly through reactive oxygen species formation. Plant Physiol Biochem. 2012;54:43–8.
